# A tale of two cities: contrasting equity of greenspace benefits in relation to PM_2.5_ exposure

**DOI:** 10.1186/s12889-025-23813-x

**Published:** 2025-10-09

**Authors:** Katherine Laura Farley, Danial Owen, Alice Fitch, David Fletcher, Janice Scheffler, Laurence Jones

**Affiliations:** 1 UK Centre for Ecology & Hydrology, Environment Centre Wales, Deiniol Road, Bangor, LL57 2UW UK; 2 UK Centre for Ecology & Hydrology, Bush Estate, Penicuik, Midlothian EH26 0QB UK

**Keywords:** Air pollution, Green infrastructure, Inequality, Deprivation, Public health, Urban planning

## Abstract

**Background:**

Urban air pollution is the second highest risk factor for non-communicable diseases. Socioeconomically deprived populations are at greater risk from related adverse health outcomes and often bear the additional burden of living in areas with higher pollution exposure. To reduce this inequality policy makers need to understand the relationship between the location of interventions and improvements in air quality. The 3–30-300 greening rule outlines guidance to improve residents’ access to trees and greenspace. No studies have yet evaluated its implications for reductions in inequalities or co-benefits beyond greenspace access. This paper explores the relationship between exposure to PM_2.5_ and socioeconomic status in Paris and Aarhus, and the potential impact of the 3–30-300 rule on health inequalities.

**Method:**

We use air quality models and socioeconomic data to calculate disparities in access to greenspace and exposure to fine particulate air pollution across subgroups using two land cover scenarios: existing greenspace conditions and potential enhancements following the 3–30 rule. The following socioeconomic data were disaggregated to residential buildings: households in poverty, age group, citizenship, employment, and education. Exposure to air pollution was calculated as the population-weighted average of PM_2.5_ concentration (µg m^−3^).

**Results:**

The relationship between socioeconomic characteristics and exposure to poor air quality differed in each city: In Paris, populations often considered more vulnerable to adverse health outcomes (unemployed, non-citizens, lower education level, lowest income) were exposed to higher concentrations of PM_2.5_, (although the relationship with income was nonlinear), whilst both older and younger populations had slightly lower particulate exposure than working age populations. By contrast, in Aarhus, populations with higher socioeconomic status (and by association, lower vulnerability) in terms of income, education, citizenship and employment status, were exposed to higher concentrations of PM_2.5_, whilst children were the age group least exposed to fine particulates.

Increasing greenspace under the 3–30 had contrasting effects on inequities in each city: mitigations improved air quality for all populations in both but, in Paris, the greatest benefit was experienced by those with lower baseline exposure as well as lower vulnerability. In Aarhus larger air quality improvements were experienced by populations with the highest baseline exposure except for the income characteristic. These were not groups expected to be at greater risk of adverse health outcomes.

**Conclusions:**

Reductions in PM_2.5_ concentration can be produced via targeted increases in trees. The impact on public health outcomes depends in part, on the spatial distribution of socioeconomic groups. Somewhat counter-intuitively, the 3–30 rule in both cities gave greater benefit to those with lower vulnerability.

## Introduction

Air pollution is one of the greatest risks to human health: an estimated 4.2 million premature deaths are caused by ambient air pollution globally [1] and it is the second highest risk factor for non-communicable diseases worldwide [1] which include asthma and chronic obstructive pulmonary disease (COPD), cardiovascular disease (CVD) [2], and acute lower respiratory tract infections (ALRTI) [1]. Higher concentrations of air pollutants are associated with lower self-reported health [3–5] and poorer mental health, whilst long term exposure to pollutants is associated with increased risk of mental illness (depression and anxiety [6–10] cognitive development [11], and dementia [12]). These non-communicable mental and physical diseases contribute to poorer quality of life and life years lost (LYL) with their associated socioeconomic consequences.

Fine particulate matter with a diameter up to 2.5 μm (PM_2.5_) is responsible for a substantial component of this health burden, and is linked specifically to all-cause mortality, cardiopulmonary and lung cancer mortality, and low birthweight [13, 14]. All levels of PM_2.5_ concentration have harmful outcomes with a clear increase in mortality rate associated with increases in concentrations [15]. The importance of improving ambient air pollution is recognised globally with a reduction of mortality and morbidity from air pollution forming a component of Sustainable Development Goal (SDG) 3.9.1. In 2024, the European Union provisionally reduced the annual PM_2.5_ limits to 10 µg/m^3^ [13] and the WHO reduced limits to 5 µg/m^3^ in 2021 [14]. Reducing air pollution is thus important in reducing prevalence and outcomes of chronic health conditions.

### At risk populations

Adverse health effects from air pollution are greater for socio-demographic groups whose health, economic, or environmental factors increase vulnerability. Socioeconomic disadvantage is associated with poorer health outcomes including higher CVD, respiratory disease and mental illness prevalence, infant mortality, comorbidities, and higher mortality. This greater burden of existing chronic health conditions means that deprived populations are more vulnerable to the effects of air pollution [15].

As well as socioeconomic disadvantage, age has a notable influence on vulnerability. Existing comorbidities such as reduced lung function, ALRTI, IHD, stroke, and COPD mean that older people have a greater vulnerability to respiratory and cardiovascular outcomes from ambient air pollution and are at greater risk from short term exacerbation of symptoms. Children and young people (CYP) are also at greater risk due to their developing physiology.

Some ethnic groups may be more vulnerable due to variation in the prevalence of existing health conditions, for example, Black and South Asian people are more likely to have a diagnosis of CVD [4, 16]. However, some evidence shows a complex relationship [4, 5, 16] in the UK, where overall minority groups tend to have poorer self-reported health, but black African and Caribbean populations report better health than British white people [5].

### Variation in exposure

Certain populations are more likely to be exposed to higher concentrations of air pollution across multiple environments (including the home, occupational environments, neighbourhoods, and commuting) [17]. In the USA, this positive relationship between air pollution and poverty is relatively clear but the relationship in the European Union (EU) is complex [18] with greater variation in patterns between cities: some studies report that populations with low socioeconomic status (SES) are more exposed to pollutants [19, 20] whilst others indicate an inverse relationship [15]. Socioeconomically disadvantaged populations are also more likely to experience less favourable living conditions than more affluent populations [21], and neighbourhood socioeconomic status is a potential risk factor for some individual health outcomes [22]. Immigrant populations in particular may be exposed to above average concentrations of particulate matter [15]. Ethnic segregation is also important: some minority groups are more likely to live in areas with higher air pollution as well as experiencing higher deprivation.

### Intersectionality of exposure, vulnerability and socioeconomic status

There is a complex relationship between socioeconomic, environmental, and health factors, supporting the triple jeopardy hypothesis that low-SES communities face increased susceptibility to ill-health; higher exposure to pollution; and health disparities driven by environmental hazards [23]. Age and ethnicity are important determinants of risk, with older people, children and some minority ethnic groups living in disadvantaged areas likely to experience both higher exposure and to have greater vulnerability. People at the intersection of multiple characteristics are at particularly high risk of adverse health outcomes. Since both air pollution and clustering of populations with high risk factors exhibit strong spatial patterns, understanding the spatial distribution of air pollution and socio-economic status in cities can assist in planning local health service provision [16, 17].

### Potential health benefits of tree planting

Evidence from Spain suggests that reducing fine particulate pollution could lead to thousands fewer deaths per year [24]. Improving air quality can be achieved by directly reducing emissions at source, for example via regulation of vehicles and their access to urban areas, or controls on other emissions including rural sources of PM_2.5_ and its precursors such as ammonia. Alternatively, increasing the proportion of vegetation such as trees, shrubs and to a lesser extent grass and crops can reduce particulate matter present in the air [25, 26]. Although removal of air pollution by greenspace can only be a partial mitigation, green infrastructure offers a wide range of health co-benefits such as noise reduction, shading, and mental wellbeing [27, 28].

### 3–30-300 rule

Cities around the world, such as Barcelona, Denver [29] Manchester [30], are increasingly developing ambitious plans to increase the amount of green infrastructure, recognising the many co-benefits. These ambitions are typically expressed as a percentage increase from current levels of e.g. tree cover, since cities have widely varying levels of existing tree cover, but there is an ambition to aim for certain minimum standards. An approach to setting a target of ambition which is rapidly gaining traction is the 3–30-300 rule, which simultaneously aims to increase green space (with a focus on trees) and address underlying equity issues in the distribution of green space within cities. The 3–30-300 rule has three main components/targets: each workplace, school, or home should have a minimum of three viewable trees, 30% neighbourhood tree canopy cover, and be within 300 m of their nearest green space of minimum one hectare (ha) [31, 32]. Few cities meet all the components of the rule [33], but it shows potential to partially address both air quality and equity issues in urban areas due to its focus on improving tree cover across all areas of a city.

Existing studies have explored the relationship between socioeconomic characteristics and air quality in Paris [18]. Other studies have quantified the amount of air pollution removed by trees in studies in the USA [34] and UK [26, 35], and the associated reduction in PM_2.5_ concentrations due to urban tree cover, which can range from 0.1–10%. Relatively few studies have looked at the inter-relationship between socioeconomic status, air pollution exposure and the benefits of greenspace, and no studies have yet quantified the potential benefits of the 3–30-300 rules in reducing inequities associated with air pollution exposure. This paper explores the impact of such initiatives on socioeconomic groups likely to be at greater risk from the effects of PM_2.5_ due to higher prevalence of chronic health conditions usually found in these communities.

## Aims & objectives

This paper aims to evaluate equity issues for different socioeconomic groups around the potential of tree planting interventions under the 3–30-300 rule to mitigate outdoor air pollution on public health. The study compares two cities with contrasting demographics, size, and population distribution, with respect to how four socioeconomic factors (age, income, education, & citizenship) play out in terms of:

- Inequity in exposure to current levels of air pollution
- Whether the tree-planting component of the proposed 3–30-300 rule for enhanced green space will exacerbate or will improve existing inequities

## Method

In this section, we provide an overview of the data used (see “ Study sites” section) and explain how we measure the population-weighted values for both residential buildings’ average air quality and tree cover which is a key variable linked to removal of PM_2.5_ pollution by vegetation (see “ Data” section).

### Study sites

This study takes a case study approach, comparing the effects of the rule in the context of two cities with contrasting demographies: Paris in France and Aarhus in Denmark. These cities have differing histories of migration and urban development, as well as the spatial distribution of residential properties. They have also been used in a recent assessment of the potential to implement the 3–30-300 greening rule [33], providing readily available spatial scenario data on tree cover to apply in this analysis.

#### Paris

Paris city region has a population of 12.1 million of which 2.2 million reside in the central City of Paris neighbourhoods. It is the second most multicultural city in Europe [36] and has spatial differentiation of income subgroups, with the north-east predominantly more deprived and the west and wider satellite urban areas being more affluent. In the second decade of the twenty-first century, there was a rise in immigration and there are high levels of segregation for non-European immigrants [37]: due in part to the historical location of public housing projects, industry and jobs, migrants generally settle in suburbs peripheral to the central City of Paris [38]. Urban disadvantage and immigration are strongly linked in the city and there is high income inequality [39]; a history of social mix policies to combat segregation has been deemed only partially successful [40]. In 2015, 1100 premature deaths (32.5% of all air pollution related deaths) in metropolitan Paris were attributable to ambient PM_2.5_ and ozone from transportation tailpipe emissions [41]. The city has implemented several initiatives to address the limited amount of green space per capita, improve air quality, and alleviate associated environmental pressures. These include the introduction of Low Emissions Zones in 2015, 2016 & 2017, and Euro 5 regulation, as well as emergency speed restrictions and free public transport during pollution events [42]. Other interventions include plans to plant 170 000 trees across the city in between 2020 and 2026 to cut noise, air pollution and tackle global warming [43], and to increase green roof coverage by 100 hectares [44].

#### Aarhus

Aarhus is the second largest city in Denmark, with a relatively young population. The population of Aarhus rose rapidly over the past two decades to 355 238 in 2021, 9.53% of this population are immigrants and just 15% are over 64 years old [45]. This population increase has led to an increase in population density. The city has a commitment to nature-based solutions (NBS) and ecosystem services (ESS) with an ambition to double the city’s total nature area by 2023 [33] and increase forested land by 60% by 2030 [46].

### Data

Datasets used in this study include high-resolution land cover, air quality model outputs, disaggregated building-level population, and socioeconomic/demographic data (Table 1). The high-resolution (5m) land cover data used was obtained from Owen et al. [47], derived from Knopp [48, 49]. The baseline PM_2.5_ concentration data used in combination with air quality modelling in this study was obtained from Hammer et al. [50, 51]. For Paris, all social data was obtained from France’s annual census survey for 2020 at the IRIS level, the smallest statistical unit (Table 1). In Aarhus, the social data was made available by Statistics Denmark, processed by Geomatics and is available at the district and 1 km grid resolution [52].

**Table 1:** Data and variables

Variable	Sub-categories	Resolution (Paris)	Resolution (Aarhus)
Land cover			
Current landcover (2015, 2017)	-	5 m	5 m
Air quality PM_2.5_			
Satellite data (2019)	-	1 km	1 km
Building-level population			
Disaggregated population	-	Building	Building
Social data			
Population	-	IRIS	District
Age	< 18	IRIS	District
	18–64		
	≥ 65		
Citizenship	Citizen	IRIS	District
	Non-citizen		
Employment	Employed	IRIS	District
	Unemployed		
Education	Primary	IRIS	District
	Secondary		
	Tertiary		
Income	Bottom Quintile	IRIS	1 km
	Middle Quintiles		
	Top Quintile		

#### The 3–30-300 rule

The full methodology behind how the 3–30-300 rule was implemented for each city is outlined in Owen et al. [33] with the resulting landcover available from Owen et al. [47]. This interpretation differs slightly from the original ideas of Konijnendijk [31] in that all green and blue space is included as a component of the neighbourhood 30% tree cover target. In this study, we only analyse the impacts of the ‘3’ and ‘30’ components of the 3–30-300 scenario, i.e. every residential building to have at least three viewable trees (defined as two 5 m tree raster cells within 30 m) and every neighbourhood to have at least 30% green (tree canopy) and blue space cover.

#### Air quality modelling

To investigate the impact of current tree cover and the new 3–30 tree planting on PM_2.5_ concentrations, a statistical meta-model in the form of a regression equation for each city was created from the outputs of the atmospheric chemistry transport model EMEP-WRF.

The use of meta-models has distinct benefits in allowing rapid assessment for policy purposes or exploring multiple scenarios, without re-running complex atmospheric chemistry models each time. Examples of applying meta-models derived from the atmospheric chemistry transport model EMEP-WRF, which is described in more detail below, include use within an integrated modelling platform evaluating government policies in Wales [53], and a comparison of greenspace benefits in selected cities across the world [25, 26, 53, 54]. EMEP-WRF [55–57] is the European Monitoring and Evaluation Programme (EMEP) MSC-W, coupled with the Weather and Research Forecasting model as a meteorology driver. The model set up here used EMEP version rv4.45 and WRF version 4.2.2 [58], nudged towards ERA5 re-analysis data [59]). Anthropogenic pollution emission data from the EMEP emission inventory Centre for Emission Inventories and Projections (CEIP, http://www.ceip.at/) were used as inputs. EMEP calculates chemical interactions, exchanges and transport for 80 pollutants, with pollutant removal via dry deposition depending on the land cover class [57]. 2018 data were used for the meteorology and the input emissions. EMEP-WRF has been validated for investigating impacts of air pollution changes on health by Lin et al. [60] and applied in other studies quantifying the air pollution removal by vegetation based on full EMEP-WRF runs [25, 26].

For the EMEP modelling, specific scenarios for each city were used to build the regression equations. These entailed an EMEP scenario with current urban vegetation and a counterfactual EMEP scenario where all urban trees were removed and replaced with bare soil. The change in pollution concentration due to PM_2.5_ deposition to leaf surfaces is calculated by difference between the scenarios. The bare soil counterfactual was used to calculate the pollution removal by current urban trees. This allows derivation of a flexible meta-model which can be used to estimate the impact of varying additions or reductions in tree-cover under a range of policy scenarios. Previous modelling runs have established that the effect of tree cover on pollution concentration is approximately linear, with the main variation governed by vegetation type and initial pollutant concentration [53, 54]. The grid cell size within the city domains was 3 × 3 km. In this study we focused only on developing meta-models for the pollution concentration change, rather than quantity of pollution removed, since the PM_2.5_ concentrations directly influence population exposure and health outcomes. The meta-model equations were derived by extracting gridded outputs of the models and developing a regression model based on an understanding of key input parameters (proportion of tree cover, initial PM_2.5_ concentrations) refined in previous studies [53]. The meta-model is based on a full-year run of EMEP-WRF, so it incorporates hourly variation in meteorology and pollutant interactions as well as seasonal influences on vegetation which are all represented within EMEP-WRF.

The regression equations give a percentage change in concentration associated with a given proportion of woodland cover, which can then be applied to the background concentration to estimate the actual change in concentration (which will be a negative value, representing the reduction in pollution concentrations due to the woodland). The equation derived for Aarhus (R^2^ = 0.754) is provided in Eq. (1) and that for Paris (*R*^2^ = 0.689) in Eq. (2):

1 $$Aarhus pcPM=\left(-3.9134*woodfrac\right)-0.1636$$

2 $$Paris pcPM=\left(6.0425*woodfra{c}^{2}\right)-\left(8.9865*woodfrac\right)-0.8939$$

where woodfrac is the proportion of woodland within a grid cell (ranging from 0 to 1), and pcPM is percentage change in PM_2.5_ concentration (% µg m^−3^).

The regression equations were applied to calculate PM_2.5_ concentration change for current landcover and for landcover under the 3–30-30 scenario for this study as follows. Proportion of woodland was calculated for each 3 km grid cell and used to predict the change in PM_2.5_ concentration for current landcover and the 3–30 rule. Analogous to bias-correction approaches in climate scenario modelling, we then calculated the absolute difference in PM_2.5_ concentration relative to the EMEP baseline PM_2.5_ concentration from the EMEP model outputs at 3 km resolution for each landcover scenario. These differences were bilinearly resampled to 1 km and applied to a finer resolution (1 km, data for 2019) global dataset of PM_2.5_ concentration [50], used as the baseline air quality data in this study, to obtain PM_2.5_ concentrations under current landcover and the 3–30-300 scenario for each city.

#### Social data classification

The social data used in this study comprised demographic information (age group and citizenship), and components of socioeconomic status (SES) (employment status, educational attainment, and income) [61]. Age groups were classified into three classes (below 18; 18–64; 65 and above), citizenship status into two classes (citizen; non-citizen), employment status into two classes (employed; unemployed), educational attainment into three classes (primary; secondary; tertiary), and income into three classes (bottom quintile; middle quintiles; top quintile). Of the above, citizenship and employment status were already classified as such in the raw data. Educational attainment was manually aggregated to widely used categories: primary, secondary, and tertiary. The remaining variables were classified based on evidence from the literature on population sensitivity to air pollution. For example, those under 18 are still developing physiologically while those above 65 are more likely to have co-morbidities. Income was partitioned into three classes, to differentiate the locations within the highest and lowest incomes.

#### Disaggregation of socio-demographic characteristics to building-level

The social variables in Paris and Aarhus were disaggregated to building level, by first calculating the proportion of the population of each variable (e.g. employed) relative to the total population of the respective unit (e.g., IRIS), then multiplying the proportion by the building-level population.

### Population-weighted averages

#### Air pollution exposure (PM_2.5_)

In each city, we calculate a single population-weighted average PM_2.5_ concentration (µg m^−3^) for each population subgroup (*a*), for both the baseline and the 3–30 component of the 3–30-300 rule scenarios, using disaggregated building-level population data (see “ Disaggregation of socio-demographic characteristics to building-level” section). This is calculated by multiplying each building’s (*u*) subgroup population by its PM_2.5_ concentration, summing these values for all buildings, and then dividing this sum by the total subgroup population (Eq. 3). Each building’s PM_2.5_ concentration was extracted using the point sampling tool (Borys Jurgiel, v0.5.4) in QGIS v3.4.

3 $$Population weighted P{M}_{2.5 }{(\mu g/{m}^{3})}_{a} = \frac{{\sum }_{u=1}^{n}Populatio{n}_{a,u} \times P{M}_{2.5} {\left(\frac{\mu g}{{m}^{3}}\right)}_{u}}{{\sum }_{u=1}^{n} Populatio{n}_{a, u}}$$

#### Trees

To help explain changes in air quality between the baseline and 3–30-300 scenario, we calculate the population-weighted average tree cover (m^2^) within 300 m for each building. 1Once we have the tree cover (m^2^) for each building, we then calculated the population-weighted average for each population subgroup (*a*) for the same spatial units as used for population-weighted PM_2.5_ concentration for both the baseline and 3–30-300 scenario. This was calculated by multiplying each building’s disaggregated subgroup population by its tree cover (m^2^), summing these values for all buildings, and then dividing this sum by the total subgroup population (Eq. 4).

4 $$Population weighted tree cover {({m}^{2})}_{a}= \frac{\sum_{u=1}^{n}{Population}_{a,u}\times {Tree cover ({m}^{2}) }_{u}}{\sum_{u=1}^{n} {Population}_{a, u}}$$

## Results

### Aarhus

#### Baseline exposure

Average baseline PM_2.5_ concentration in Aarhus was 10.64 µg m^−3^ (Table 2). The socioeconomic group exposed to the poorest quality air was the highest income quintile with a difference of 0.12 µg m^−3^ from the group with the best air quality (lowest income quintile). Average concentration for the lowest income quintile was 10.57 µg m^−3^ rising to 10.67 µg m^−3^ for the middle quintiles and to 10.69 µg m^−3^ for the highest income quintile. Thus, areas with the highest average income level in Aarhus had the greatest exposure to PM_2.5_ at baseline.

**Table 2:** Population weighted value, and absolute change of PM_2.5_ (µg m^−3^) concentration for the baseline and 3–30-300 land cover scenarios across each population subgroup in Aarhus

		**PM**_**2.5**_** concentration (μg m**^**−3**^**)**		
Category	Sub-Category	Baseline	3–30-300	∆ PM_2.5_
Age	< 18	10.61	10.58	−0.028
	18–64	10.65	10.61	−0.038
	≥ 65	10.65	10.62	−0.030
Citizenship	Citizen	10.65	10.61	−0.035
	Non-Citizen	10.58	10.55	−0.034
Education	Primary	10.61	10.58	−0.033
	Secondary	10.64	10.60	−0.036
	Tertiary	10.66	10.63	−0.038
Employment	Unemployed	10.63	10.60	−0.036
	Employed	10.65	10.62	−0.036
Income	Bottom quintile (lowest income)	10.57	10.53	−0.042
	Middle quintiles	10.67	10.64	−0.034
	Top quintile (highest income)	10.69	10.67	−0.020
Total		10.64	10.61	−0.035

Smaller variations were identified between other population groups but the pattern of some more vulnerable populations being exposed to lower concentrations of PM_2.5_ persisted. For example, children were exposed to relatively lower concentrations (10.61 µg m^−3^) than older people (10.65 µg m^−3^) or the working age population (10.65 µg m^−3^). Non-Danish citizens were exposed to lower concentrations then those with citizenship (10.58 µg m^−3^ vs 10.65 µg m^−3^), and more educated populations had a higher exposure (10.66 µg m^−3^) than those with less education, with those with only primary education having the lowest exposure (10.61 µg m^−3^).

#### Impact of 3–30-300 mitigations

Mitigations from the 3–30 rule had a small impact on levels of PM_2.5_ across all populations, with an average overall reduction of −0.035 µg m^−3^ (Table 2). The greatest improvement for a single socioeconomic group was for people in the lowest income quintile (−0.042 µg m^−3^), i.e. those expected to be most vulnerable. From one perspective, the impact of the intervention is inequitable across income quintiles, since areas with the worst existing air quality (high income quintile) received the smallest improvement (−0.020 µg m^−3^). This leads to a widening in the current difference between low income and high-income areas from 0.12 µg m^−3^ to 0.14 µg m^−3^. However, since the lowest income group received the most benefit from 3–30-300, this may be seen as positive.

For some characteristics, the largest relative improvement was observed for those populations which had the highest baseline exposure, but which are likely to be less vulnerable to adverse outcomes. For example, for age, the largest improvement was found for the working age population (−0.038 µg m^−3^). Similarly, for education, the group which experiences the greatest improvement in terms of absolute change of PM_2.5_ was inhabitants with a tertiary level education, with a reduction of −0.038 µg m^−3^ relative to the average reduction of −0.033 µg m^−3^ for those with primary level education. For other socioeconomic categories there was very little or no variation in the absolute or relative change of PM_2.5_: employed and unemployed categories both saw a reduction of −0.036 µg m^−3^ and the difference between citizens and non-citizens was 0.001 µg m^−3^. Thus, although the air quality for all residents improved with the modelled intervention, there was very little narrowing of the gap in terms of employment or citizenship status.

Overall, therefore, more disadvantaged and, by proxy, more-vulnerable populations in Aarhus were exposed to lower levels of air pollution than wealthier or less-vulnerable populations. However, the 3–30 rule had variable outcomes for different vulnerabilities, further widening the gap in exposure of wealthy vs lower incomes, but slightly reducing disparities of exposure across education level, and having no effect on disparities among employment or citizenship.

The average tree cover within 300 m of subgroups (Table 3) broadly reflected changes in PM_2.5_ concentration for education and citizenship categories. At baseline, middle income, working age, employed, and tertiary education populations had the least tree cover and highest income the most. These groups benefitted from the greatest increases in tree cover thus tree planting in Aarhus under the rule led to more equitable tree coverage across all categories.

**Table 3:** Population weighted average and absolute change in tree cover (ha) within 300 m of residential buildings, for existing conditions and the 3–30-300 land cover scenarios in Aarhus

		**Tree Cover (ha)**		
Category	Sub-Category	Baseline	3–30-300	∆ in area
Age	< 18	3.22	3.52	0.30
	18–64	2.99	3.49	0.50
	≥ 65	3.22	3.52	0.31
Citizenship	Citizen	3.06	3.50	0.44
	Non-Citizen	3.18	3.57	0.39
Education	Primary	3.18	3.55	0.37
	Secondary	3.06	3.52	0.45
	Tertiary	3.04	3.52	0.48
Employment	Unemployed	3.08	3.51	0.43
	Employed	3.01	3.49	0.48
Income	Bottom quintile (lowest income)	3.22	3.64	0.42
	Middle quintiles	2.80	3.31	0.51
	Top quintile (highest income)	3.70	3.88	0.18
Total		3.07	3.50	0.43

### Paris

#### Baseline exposure

Paris had overall ~ 50% higher levels of PM_2.5_ concentrations under baseline conditions than Aarhus, with a population-weighted average of 15.30 µg m^−3^ across all residential buildings (Table 4). Citywide concentrations of PM_2.5_ exceeded the EU threshold of 10 µg m^−3^ [15]. Variation in exposure between different populations was small (maximum of 0.24 µg m^−3^) for all characteristics, although this is two times the difference between comparative groups in Aarhus. As in Aarhus, the greatest variation (0.24 µg m^−3^) in baseline concentrations in Paris was between income categories. However, unlike Aarhus, Table 4 shows that for income both the lowest income group and the most affluent populations have a relatively higher exposure to poor air quality than the middle-income group, with this exposure greatest for the low-income group. This U-shaped relationship is also present for education, where the greatest exposure to PM_2.5_ is for the lowest and highest levels of education.

**Table 4:** Population weighted value, and absolute change of PM_2.5_ (µg m^−3^) concentration for the baseline and 3–30-300 land cover scenarios across each population subgroup in Paris

		**PM**_**2.5**_** Concentration (μg m**^**−3**^**)**		
Category	Sub-Category	Baseline	3–30-300	∆ in PM_2.5_
Age	< 18	15.28	15.18	−0.099
	18–64	15.32	15.18	−0.136
	≥ 65	15.28	15.14	−0.133
Citizenship	Citizen	15.29	15.16	−0.130
	Non-Citizen	15.38	15.27	−0.113
Education	Primary	15.34	15.25	−0.093
	Secondary	15.28	15.18	−0.098
	Tertiary	15.31	15.14	−0.169
Employment	Unemployed	15.35	15.23	−0.124
	Employed	15.31	15.17	−0.140
Income	Bottom quintile (lowest income)	15.46	15.41	−0.059
	Middle quintiles	15.22	15.09	−0.132
	Top quintile (highest income)	15.33	15.11	−0.211
Total		15.30	15.18	−0.127

In Paris, under current landcover, some of the most vulnerable populations were exposed to the highest concentrations of PM_2.5_. Table 4 shows that this is the case for income, employment, education, and citizenship status. In contrast, for age, more vulnerable populations (under 18 and over 65), had a lower baseline exposure level (−0.04 µg m^−3^) relative to the working age population.

#### Impact of 3–30 mitigation

The impact of mitigation on overall PM_2.5_ concentrations in Paris was small (−0.127 µg m^−3^) but more than double that in Aarhus. In direct contrast with Aarhus, the largest improvements in air quality were found for the highest income quintile (Table 4). The difference in air quality between the highest income and lowest income quintiles widened from a gap of 0.13 µg m^−3^ to 0.30 µg m^−3^. This pattern was also observed for employment, citizenship, and education, where less vulnerable populations (i.e., the employed, citizens, and those with tertiary education) all benefited the most from the intervention with a greater decrease in exposure to PM_2.5_ relative to more vulnerable populations, despite having lower baseline exposure. This indicates that despite leading to overall air quality improvement, inequity in exposure is *increased* by the mitigations.

The 3–30 rule had a greater impact on working age adults and older people than for children and young people. Although this reduces inequality of exposure across the subgroups, especially for those of working age with an existing vulnerability, given the higher vulnerability in children due to their developing physiology, the potential for public health benefits is more limited. The greatest air quality improvements were also found for those with the highest education level, for the employed population, and for citizens, thus increasing the exposure differential to the detriment of the less-educated, or more-deprived or non-citizens respectively.

Overall, in Paris the PM_2.5_ exposure at baseline was greater for some vulnerable groups (non-citizens, the least wealthy, the unemployed) but showed non-linear relationships for some aspects (age, where greatest exposure was in the middle age-group, and education level where greatest exposure was those in the least and in the most educated groups). Urban greening under the 3–30 rule exacerbated inequality of exposure by benefitting the most income rich, French citizens, the employed and the most highly educated.

In Paris, tree planting under the rule did not reduce inequalities in tree coverage across categories, and actually widened inequality across the income categories where the richest quintile had the greatest initial tree cover, but also received the most additional tree cover under the 3–30 component of the 3–30-300 rule. The greatest improvements in air quality for these less vulnerable populations can be explained by the greater change in tree cover (Table 5). The increase in tree cover generally reflects the reduction in PM_2.5_ for those populations and explains why the improvement in AQ is found in the areas where people already have good AQ.

**Table 5:** Population weighted average and absolute change in tree cover (ha) within 300 m of residential buildings for existing conditions and the 3–30-300 land cover scenarios in Paris

		**Tree cover (ha)**		
Category	Sub-Category	Baseline	3–30-300	∆ in area
Age	< 18	4.72	5.01	0.28
	18–64	4.52	4.93	0.41
	≥ 65	4.73	5.10	0.38
Citizenship	Citizen	4.63	5.02	0.39
	Non-Citizen	4.42	4.75	0.34
Education	Primary	4.59	4.84	0.25
	Secondary	4.74	5.01	0.27
	Tertiary	4.47	5.01	0.54
Employment	Unemployed	4.48	4.85	0.36
	Employed	4.52	4.95	0.44
Income	Bottom quintile (lowest income)	4.55	4.66	0.12
	Middle quintiles	4.57	4.96	0.39
	Top quintile (highest income)	4.69	5.42	0.73
Total		4.60	4.98	0.38

## Discussion

The 3–30 component of the 3–30-300 rule and the introduction of new trees and grassy areas aims to reduce the disparities in visibility, presence, and access to urban green space. This study demonstrates that some reductions in PM_2.5_ concentration for all populations can be achieved via increased tree planting applying the 3–30 urban greening rule. However, while this rule aims to reduce inequity, in practice our data show that it has the potential to worsen existing inequalities. Differences in initial exposure and the benefits of greening were dependent on existing socioeconomic demographic patterns in both case study cities. In Aarhus, existing disparities in exposure to PM_2.5_ tended to favour the more vulnerable and although greening benefitted all populations, it either emphasised these differentials (i.e. gave greater benefit to the most vulnerable) or had little impact on existing inequalities. By contrast in Paris, existing disparities were complex but tended to show higher exposure among the most vulnerable populations. The 3–30 rule improved air quality for all populations in Paris, but the improvements tended to favour the least-vulnerable rather than the most-vulnerable as found elsewhere in France [18, 24].

This variation in impact is likely due to both residential locations of different socioeconomic groups, and variation in opportunities for tree planting in each city which are dependent on building distribution. Paris’ socio-economic variation follows common urban patterns with deprivation concentrated in previously industrial peripheral neighbourhoods with low tree cover, away from the cultural and financial districts situated in central areas. The patterns in Aarhus are due largely to clusters of very high-income households in high quality, high cost, but dense, housing in the city centre where there is little open or green space, but also high density of consumer-related transport.

Whilst, broadly speaking, more affluent neighbourhoods tend to have more trees, cities like Paris often have concentrations of high quality, high-cost housing located in city centres close to cultural and business centres. Building density in these central areas means that existing tree numbers are low, and air quality poorer. For example, high nitrogen dioxide concentrations in Paris centre are attributable to emissions from the movement of goods and commuter transportation [15] needed to support cultural and financial services in the centre [18]. Opportunities for increasing tree cover here are restricted by high building density, in contrast to peripheral neighbourhoods which have greater opportunity for increases in tree cover.

### Implications for public health

Long-term exposure to poor air quality has been associated with premature deaths estimated to be ∼55,000 annually across France [62]. The theoretical modelling of the 3–30 rule benefitted all populations, but these exacerbating factors meant that in Aarhus it benefited some of the least vulnerable populations to a greater extent (citizens and highly educated) and others (higher income quintile and working age people) to a lesser extent. Likewise, in Paris, although the relationship between socioeconomic status and air pollution exposure was non-linear, the rule gave the most benefit to the least vulnerable populations who also had the lowest exposure at baseline, thus exacerbating socioeconomic inequalities.

However, the impact of AQ on health inequalities is likely exacerbated by the combined effects of multiple risks: some subgroups are more vulnerable to poor health outcomes due to higher rates of existing chronic health conditions. For example, in Denmark, refugees living in disadvantaged areas are at greater risk of cardiovascular events than the general population [63], alongside exposure to other environmental risks [18].

To reduce the excess burden on these groups and maximise the health impact, mitigations to reduce environmental pressures should target the most exposed vulnerable populations. The effect of urban tree planting has a potential role in a multifaceted strategy to improve air quality related health outcomes [64]. The potential standalone impact of this component of the 3–30-300 rule on AQ is small. However, the public health co-benefits such as mental health, shading, and noise reduction, in combination with ecosystems services benefits including flood reduction, carbon sequestration and biodiversity [28], increase the value of new trees in urban areas. Trees can be seen as public health assets and prioritised as an intervention to address multiple public health and wider environmental pressures. Potential limitations to the benefits of the rule also include changes to the socioeconomic status of the residents due to neighbourhood desirability, rising property prices [65], crime and injury risk should also be acknowledged.

### Location of trees

Increases in tree numbers do not always correct for disparities in exposure to PM_2.5_, because of spatial variation in population, air quality and tree cover. In Paris, the widening of inequalities in PM_2.5_ exposure after the introduction of new green space may be attributed to this complex relationship. The 3–30-300 interventions take into account both wider greenspace as well as tree cover, and do not consider existing exposure to PM_2.5_. Therefore, some less vulnerable populations receive the biggest increase in tree cover as they have a smaller extent of urban green and blue space despite having better existing air quality. In Paris this further benefitted the richest income group while in Aarhus this further benefitted the poorest income group.

While the meta-model described here only calculates PM_2.5_ removal by trees, they are typically at least five times as effective as grass at removing particulate matter from the air [35]. Therefore the'3'and'30'components of the 3–30-300 rule, which focus solely on adding new trees, are likely to have a larger impact on air quality than the'300'component which is mainly achieved by adding large areas of public greenspace, assumed to be predominantly grassland in Owen et al. [33]. Further work could refine this approach with additional meta-models to calculate pollution removal by urban grassland.

This theoretical simulation of urban regreening does not account for the complexity of implementation or the real-world consequences of replacing artificial land cover with trees [33]. A more substantial increase in street trees is needed in city centres as well as the repurposing of buildings [33]. In Paris centre, more artificial surfaces need to be regreened than in suburbs, due to the lower availability of grassy or bare soil areas here. This offers an opportunity to further reduce emissions through a reduction in transportation infrastructure (such as car parking and traffic lanes). This would be more beneficial in Paris due to the reliance on mineral surface replacement: in Paris, the proportion of mineral surface that would need to be reduced (nearly 95% of land being resurfaced) is greater than in Aarhus (approximately one third of the resurfaced land) [33]. Additionally, the creation of small (pocket) parks in urban centres offers easier access to greenspaces for inhabitants and thus the physical and mental health benefits associated with these [33]. However, these consequences themselves present implementation barriers and a greater need for behaviour change interventions and the involvement of communities in decision making processes.

A co-production approach to greenspace design could maximise engagement and impact on health. Co-production provides an opportunity to ensure regreening works alongside infrastructure and an educational role to support behaviour change. In Paris there has been a recent movement towards devolution of power and participatory approaches have led to new public gardens and pedestrian areas [27].

The effect of urban tree planting has a potential role in a multifaceted strategy to improve air quality related health outcomes [64]. The impact on air quality demonstrated by this theoretical model is very small, and unlikely to reduce mortality rates significantly as a standalone intervention. However, the public health co-benefits such as mental health, shading, and noise reduction, in combination with ecosystems services benefits including flood reduction, carbon sequestration and biodiversity increase the value of new trees in urban areas.

## Limitations

Variation between our study sites in the availability, classification, units, and spatial scales of data leads to a lack of standardised data at granular, subnational level, and the need for proxy variables (e.g. citizenship for ethnicity, income for deprivation). Variable structure also affected interpretation: for example, for age, citizenship, employment status, and education, we calculated the population-weighted value for each class across all spatial units. However, unlike age, citizenship, and employment status, we only have a single median value for income per spatial unit. This means that the population weighted value for income reflects weighted PM2.5 concentrations only in areas with specific income values, leading to more extreme values relative to others where the average is calculated across the entire city. All modelling has an inherent degree of uncertainty, as models are an abstraction of reality. PM_2.5_ concentrations for this analysis were generated from a meta-model approach, developed initially at 3 km resolution. As such, there is a degree of uncertainty with predicted concentrations for a given location. The outputs of the underlying EMEP model are validated against measured concentrations at municipal air quality monitoring stations, but local variation will cause noise around these spatial predictions. In large part, our approach minimises this by applying difference between model runs to an independent finer resolution modelled layer of PM_2.5_ concentrations produced by Paris. While the uncertainties in that external modelled data are unknown, it is likely that the uncertainty in our estimates are likely to be greatest around the calculations produced by the meta-models. The accuracy of disaggregated population data will be another area of uncertainty. On aggregate however, the model findings are likely to hold, since uncertainty in the predictions for individual grid-cells, will be smoothed when these are aggregated to city-level population weighted averages, assuming errors are uniformly distributed.

Small changes in PM_2.5_ concentration per se are unlikely to have a direct health impact on an individual. However, the epidemiological relationships linking health impacts to particulate matter concentrations assume a linear relationship and have not to-date identified lower-bound thresholds for effects. Thus, any exposure to PM is considered potentially harmful. Therefore, small changes in concentration experienced by large numbers of people may potentially still result in some adverse impacts. Since these are annual averages, they hide temporal variation in exposure, meaning that at some times of the year the difference in exposure will be much greater, potentially more directly influencing health outcomes than the small differences in average concentrations would suggest. Of more consequence is that these are average changes over a 1 × 1 km grid square. This smooths out considerable variation that occurs within a grid-square at finer resolutions, e.g. close to point-source traffic emissions where exposure, and changes in concentration as a result of pollutant removal by vegetation, will be greater. Lastly, this can be considered as an under-estimate of the effects of vegetation since it only covers the change in exposure that comes from the removal of particulates by deposition onto leaf surfaces. It does not take into account barrier effects potentially provided by the same vegetation which can lead to lower pollutant exposures immediately downwind [66].

## Conclusion

Urban trees should be regarded as public health assets for their direct impact on air pollution, noise and mental health alongside indirect health improvements, and the long-term global benefits from climate change mitigation. However, many regreening programmes fail to accommodate social and health vulnerabilities [67]. While the 3–30-300 rule aims to reduce inequalities of access to green space and its co-benefits, in reality, a simple application to bring all areas of a city into compliance with the rule may exacerbate existing health inequalities. Therefore, to maximise improvements to public health by improving AQ for the most vulnerable, attempts to reduce exposure need to be targeted, both in areas with higher exposure, and with larger vulnerable populations.

To maximise the impact of small reductions in PM2.5 identified here, greening initiatives also need to be implemented in conjunction with other mitigations such as extensions to Low Emissions Zone perimeter or modifications to residential properties to reduce emissions of air pollutants as well as exposure via the built environment. The effectiveness of initiatives such as park-based tree-planting could be increased through parallel behaviour change and infrastructure to improve access to and engagement with these spaces. An inclusive approach to urban planning which incorporates communities in decision making processes would maximise effectiveness.

There is a lack of evaluation of the equity implications of air pollution interventions in urban areas. Future research should consider how urban and public health planners can create integrated planning approaches which incorporate tree planting in public health outcomes. Evaluations of urban tree planting should adopt a multidisciplinary approach and integrate multiple benefits to ecosystem services as well as the social and health value added. Further exploration is also needed into the combined relationship between blue space, green space, and air quality and in the relative benefits of targeted planting for multiple outcomes.

## Data Availability

https://www.regreen-project.eu/urban-living-lab/aarhus/. Knopp JM. High resolution land cover 2015 Aarhus, Denmark. In: Zenodo, editor. IEEE Journal of Selected Topics in Applied Earth Observations and Remote Sensing2021. Knopp JM. High resolution land cover 2017 Ile-de-France [Data set]. In: REGREEN—Fostering nature‐based solutions for smart gahutiEaCHGN, editor. 2022.

## Abbreviations

ALRTI: Acute Lower Respiratory Tract Infection
AQ: Air Quality
BGS: Blue and Green Space
CVD: Cardiovascular Disease
COPD: Chronic Obstructive Pulmonary Disease
DALY: Disability Adjusted Life Year
ESS: Ecosystem Services
IHD: Ischemic Heart Disease
LYL: Life Years Lost
NBS: Nature Based Solutions
NHS: National Health Service
SDG: Sustainable Development Goal
SES: Socioeconomic Status
WHO: World Health Organisation
